# A Case of Small Bowel Obstruction and Enterocutaneous Fistulation Resulting from a Mesenteric Haematoma following Blunt Abdominal Trauma

**DOI:** 10.1155/2017/7639265

**Published:** 2017-11-26

**Authors:** Matthew North, Nicholas Aveyard, Oyeniyi Diya, Jeremy Berger, Maitham Al-Whouhayb

**Affiliations:** Department of General Surgery, Barnet and Chase Farm Hospitals, Royal Free London NHS Foundation Trust, London, UK

## Abstract

A 23-year-old male with a history of previous abdominal surgery was involved in a road traffic accident. He was discharged after initial assessment but represented several days with small bowel obstruction secondary to a mesenteric haematoma. He underwent resection and recovered well but represented later on the day of discharge with a leaking surgical wound consistent with an enterocutaneous fistula. This was managed conservatively and closed spontaneously after ten days. This case serves to highlight that adhesions from previous surgery can tether the small bowel causing mesenteric injury following blunt-force trauma. It also demonstrates that postoperative ileus can result in an enterocutaneous fistula that has the appearance of an anastomotic breakdown but which resolves more rapidly.

## 1. Case Report

**Figure 1:**
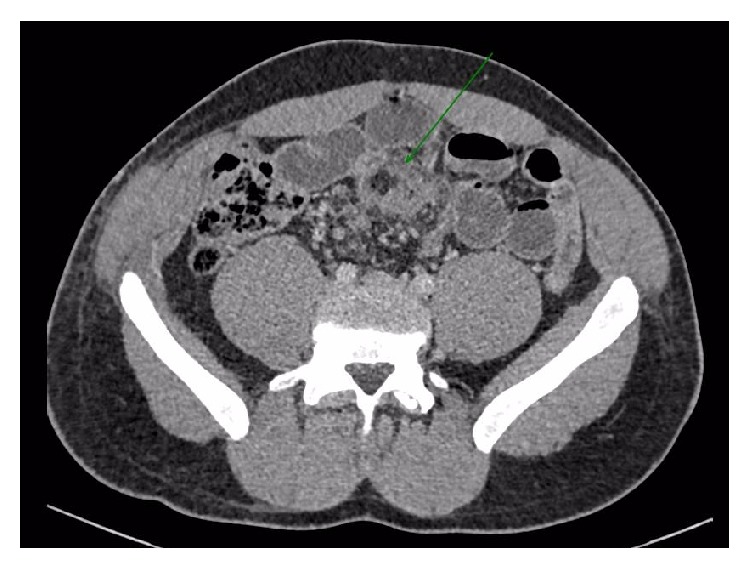
Contrast enhanced axial CT image at the time of injury demonstrated high attenuation haematoma within the small bowel mesentery (arrow).

A 23-year-old male was taken to a general hospital after the car he was travelling in collided with a tree at fifty miles per hour. CT demonstrated free fluid in the pelvis, likely from a mesenteric bleed, but no free air and no solid organ damage (Figure 1). He was transferred to a major trauma centre where he was managed conservatively and discharged the next day.

Five days later he represented to a third hospital with severe abdominal pain. A repeat CT showed an increase in the volume of pelvic free fluid and he was immediately taken to theatre for exploratory laparotomy. This revealed a mid-small bowel point of obstruction caused by adhesion to a mesenteric haematoma. Extensive adhesions were evident (the patient had an appendicectomy age nine), but there was no prior history of symptoms of obstruction. Proximal adhesiolysis was performed to allow mobilisation of bowel but this was limited by extensive peritoneal contamination and patient stability; distal and pelvic adhesions therefore remained.

Although there was no full thickness or serosal injuries to the small bowel, it was completely detached from the mesentery and showed signs of ischaemia. A 20 cm section of small bowel was resected, with a side-by-side anastomosis fashioned. Histology from the resected section of small bowel showed only inflammatory and fibrotic changes compatible with trauma.

**Figure 2:**
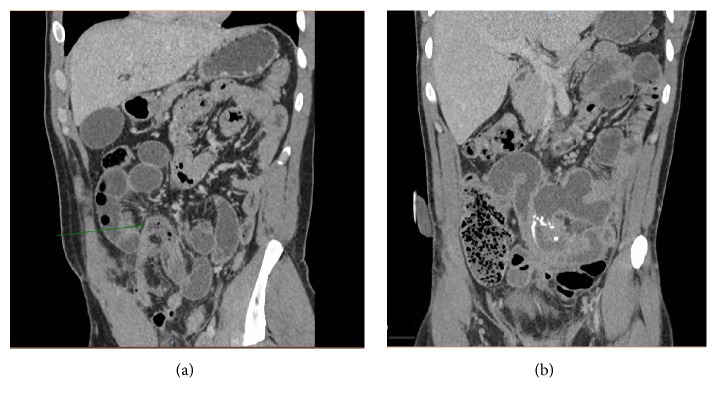
((a) and (b)) Contrast enhanced coronal CT images 5 days after trauma: there is small bowel dilatation with an acute calibre change of the small bowel indicating obstruction at the position of the previous mesenteric haematoma (arrow in (a)).

After four days in ITU, a repeat CT scan was performed as NG aspirate volumes and inflammatory markers remained high. This showed a small left iliac fossa collection with no clear features of perforation. There was small bowel dilatation with a change of calibre in the region of the anastomosis, raising the possibility of obstruction (Figures 2(a) and 2(b)). This most likely represented a paralytic ileus and it resolved without further intervention. The patient was discharged twelve days after surgery.

However, he represented later that day with a leaking abdominal surgical wound. The fluid had the appearance of undigested food, raising the possibility of an enterocutaneous fistula (ECF). A further CT scan revealed two suspected collections in the lower abdomen and pelvis, with one of these communicating with small bowel at the anastomosis and fistulating through the anterior abdominal wall. After seven days of IV antibiotics, the wound output reduced to nothing and his inflammatory markers resolved.

The patient was reviewed in the outpatient clinic five weeks later. The surgical wound had healed well, with no discharge and no signs of herniation.

## 2. Discussion

Mesenteric haematomas following blunt abdominal trauma are a recognised but rare cause of bowel obstruction and perforation (reviewed in [1]). In blunt abdominal trauma (BAT), such as that encountered in a road traffic accident, the abdominal organs most commonly involved are the liver and spleen [2]. These structures are relatively immobile, making them vulnerable to rapid acceleration and deceleration forces.

However, small bowel injury is also common in BAT [3]. In a large study at a major trauma centre, 13% of total admissions requiring laparotomy over a 5-year period were due to BAT. Of these, 13% of patients suffered mesenteric injuries [4]. Other studies have detected bowel and mesenteric injuries in 5% of BAT at laparotomy [5]. Several mechanisms of injury have been proposed, including the “fixed point” theory, whereby tangential tears occur at relatively fixed points along the bowel [3]. This theory has been supported by an historic study showing that the majority of small bowel injuries resulting from trauma occur in the proximal jejunum or distal ileum [6]. Injuries in these regions are more likely to be mesenteric damage rather than primary bowel perforation [3].

In the case described here, adhesions from the prior appendicectomy acted to tether the mid-small bowel, making it susceptible to deceleration injury and causing a mesenteric bleed. The resulting haematoma subsequently resulted in obstruction.

Diagnosis of bowel and mesenteric injuries can be difficult, and initial CT scans are often nonspecific [5]. Even short delays in diagnosis of hollow viscus injury in BAT have been shown to increase mortality [7]. Although there are few data on the proportion of “missed” injuries with delayed diagnosis, one multicentre study showed that 27% of patients had a delay in operative intervention, with a corresponding increase in mortality [8]. In the absence of specific CT findings, correlation of nonspecific CT features with clinical findings is recommended, with repeat CT scanning 6–8 hours following patient stabilisation [5].

Management of free fluid found alone on CT without organ injury is controversial, as improving imaging technology has led to an increase in conservative treatment. Several studies have attempted to provide clarity (reviewed in [9]). Banz et al. have proposed a treatment algorithm based on five simple questions, including an assessment of free fluid location and volume, as well as the clinical status of the patient [9]. Cases such as this one, where there is free fluid but no obvious organ injury, are the most difficult to manage, especially in a younger patient with a large haemodynamic reserve. Patients with a significant mechanism of injury (e.g., road traffic accident) are at high risk of a missed visceral injury and should be closely observed in the early postinjury period.

Enterocutaneous fistulae can result from a number of intra-abdominal insults, including inflammatory bowel disease and elective surgery. Trauma, however, is considered a rare cause of ECF [10]. A small-scale study has shown that nearly one-third of posttraumatic ECF heal spontaneously, without the need for further operative intervention [10]. A larger ten-year study of ECF, from a single major trauma centre, determined that 74% of ECF occurred in patients with temporary abdominal closure and were more likely to arise from the small bowel [10] (as in this case). 40% of ECF resulted from anastomotic breakdown, with an average time to spontaneous closure in this study of 34 days [11]. The rapid closure of the ECF described here is unusual and is likely due to the distal obstruction postoperatively (ileus) causing backpressure through the anastomosis, and so creating a fistula, rather than anastomotic breakdown. As the ileus resolved, this pressure dropped and was no longer sufficient to keep the fistula open, causing rapid closure.

This case highlights a rare but significant cause of small bowel obstruction following trauma, which can be delayed and easily overlooked in the context of polytrauma [12] and in the absence of serial cross-sectional imaging [5].
